# Association between the levels of perceived social support and levels of psychological distress among nurses and psychiatry doctors at the national center for mental health three years After the covid-19 pandemic: a cross-sectional study

**DOI:** 10.1192/j.eurpsy.2026.10944

**Published:** 2026-07-31

**Authors:** T. M. Chavez

**Affiliations:** National Center for Mental Health, Mandaluyong, Philippines

## Abstract

**Introduction:**

It is essential to provide clinical and policy strategies that support HCWs in the ongoing pandemics. Psychological support could include counseling services and the development of support systems among colleagues (Sharma et al., 2020). A form of coping mechanism that is necessary to decrease HCWs’ psychological distress and promote positive feelings is social support (Alnazly et al., 2021). Social support has been shown to be helpful in reducing of occupational stress and preventing common psychological distress and psychiatric symptoms among HCWs.

**Objectives:**

The study’s main objective was to examine the link between perceived social support, individual predictors, and mental health outcomes, such as depression, anxiety, and stress, among nurses, psychiatry residents, and consultants working at the National Center for Mental Health (NCMH).

**Methods:**

A total of 257 individuals participated in a survey that used both paper-based and online questionnaires to measure perceived social support using the Multidimensional Scale of Perceived Social Support (MSPSS), and mental health outcomes using the Depression, Anxiety, and Stress Scale (DASS-21). The cross-sectional survey investigated the relationship between perceived social support, individual predictors such as COVID-19 infection and marital status, and mental health outcomes. Logistic regression analyses were used to examine these associations measured at 95% confidence interval.

**Results:**

This study found that the levels of perceived social support significantly affect the levels of depression, anxiety, and stress among nurses and psychiatry residents and consultants of the NCMH.

**Conclusions:**

This study found that the levels of perceived social support significantly affect the levels of depression, anxiety, and stress among nurses and psychiatry residents and consultants of the NCMH. This study concludes that perceived social support, especially from significant others and friends, plays a crucial role in mitigating severe mental health issues among healthcare professionals. Tailored mental health interventions should focus on enhancing support from significant others and peers, addressing chronic health conditions, and providing targeted support for those affected by COVID-19. These strategies promote mental resilience and well-being in this critical workforce.

**Disclosure of Interest:**

None Declared

